# Resection of a Deep Pubic Alveolar Rhabdomyosarcoma With an Inferior Pubic Ramus Osteotomy Through a Perineal Approach: A Case Report

**DOI:** 10.1155/cro/6933702

**Published:** 2026-04-11

**Authors:** Brian Kwan, Dylan Shafer, Bonnie Balzer, Leo Mascarenhas, Daniel C. Allison

**Affiliations:** ^1^ Community Memorial Hospital, Department of Orthopedic Surgery, Ventura, California, USA, cmhshealth.org; ^2^ Cedars-Sinai Medical Center, Department of Orthopedic Surgery, Los Angeles, California, USA, cedars-sinai.edu; ^3^ Cedars-Sinai Medical Center, Department of Pathology and Laboratory Medicine, Los Angeles, California, USA, cedars-sinai.edu; ^4^ Cedars-Sinai Medical Center, Department of Pediatric Hematology and Oncology, Los Angeles, California, USA, cedars-sinai.edu; ^5^ Children′s Hospital of Los Angeles, Department of Pediatric Orthopedic Surgery, Los Angeles, California, USA

**Keywords:** alveolar rhabdomyosarcoma, case report, obturator internus, perineal approach, pudendal nerve

## Abstract

**Case:**

A 17‐year‐old male who presented with urinary retention was found to have an alveolar rhabdomyosarcoma involving the peritoneum, prostate, and bladder. After neoadjuvant chemotherapy, a residual mass in the obturator internus muscle was resected through a perineal approach and inferior pubic ramus osteotomy.

**Conclusion:**

Tumors in the obturator internus are uncommon, resulting in limited literature on approaches and technical considerations for resecting masses in this region. We present a case of successful tumor resection from the obturator internus muscle using this approach while preserving neurovascular and urogenital function with no recurrence at 24‐month follow‐up.

## 1. Introduction

Rhabdomyosarcomas can occur throughout the body but involve the genitourinary tract 13%–20% of the time [1]. Historically, these tumors had a bleak prognosis prior to modern chemotherapeutic protocols due to the morbidity of resection in this region. Current evidence suggests that chemotherapy allows for complete resection and cure rates of up to 80% [1].

Tumors involving the obturator internus muscle that require resection are relatively rare, and thus literature describing approaches to this region is limited. The described approaches primarily consist of anterior and medial approaches [2], which require extensive dissection and are associated with a greater risk of blood loss and damage to neurovascular, genitourinary, and intra‐abdominal structures.

We present a case of an adolescent male diagnosed with a pelvic alveolar rhabdomyosarcoma, who was treated with chemotherapy, radiation, and subsequent surgical resection through a perineal approach—an approach previously described only in a limited case series [3]. Furthermore, our approach incorporated an inferior pubic ramus osteotomy, with careful attention to the preservation of the pudendal nerve, to achieve a successful wide resection.

## 2. Case Presentation

A previously healthy 17‐year‐old male initially presented with several episodes of urinary retention. Urinalysis showed no evidence of a urinary tract infection. Subsequent workup with an abdominal ultrasound showed right hydronephrosis along with a 13 x 6.7 x 7.8 − cm mass adjacent to the right pelvic sidewall, causing a mass effect against the urinary bladder. Laboratory studies showed an elevated lactate dehydrogenase and C‐reactive protein.

**Figure 1:**
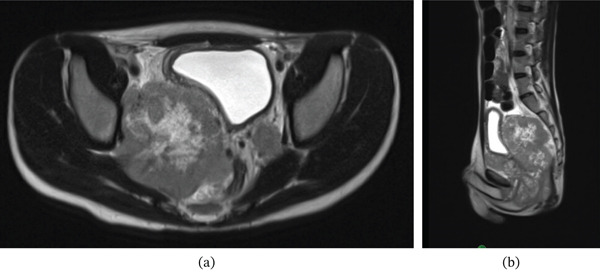
Select axial (a) and sagittal (b) MRI images demonstrating a large heterogeneous pelvic mass involving the peritoneum, bladder, and ilium.

**Figure 2:**
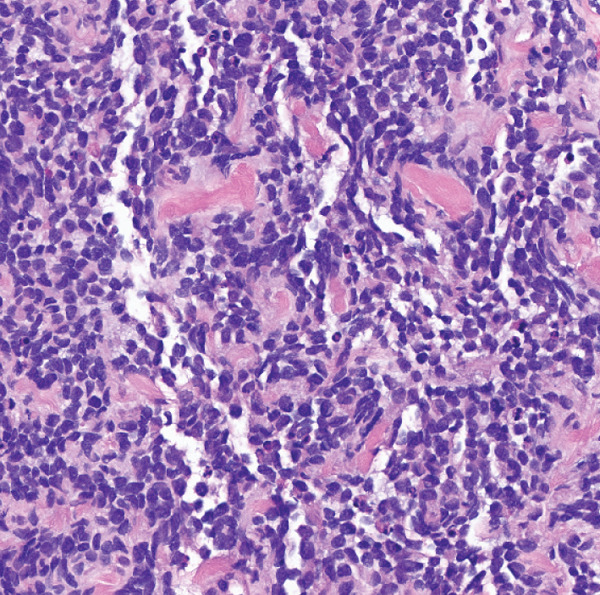
Medium power view (200X) from core biopsy showing high‐grade epithelioid rhabdomyoblasts with high cellularity.

**Figure 3:**
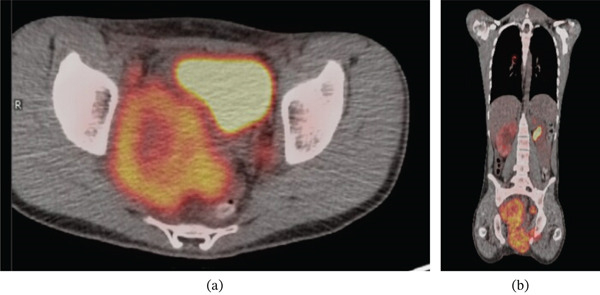
Select axial (a) and sagittal (b) PET scan images demonstrating hypermetabolic activity of the large pelvic mass.

The patient was admitted to the hospital for further workup. A magnetic resonance imaging (MRI) scan showed a large heterogeneous multicompartmental pelvic mass involving the peritoneum, prostate, urogenital diaphragm, perineum, left obturator foramen, and right iliac vessels (Figure 1). There were lymph node metastases involving the left pelvic sidewall and bilateral inguinal nodes. There was also mass effect causing right hydroureteronephrosis and mass effect onto the anus/rectum, bladder, and right sciatic notch. A computed tomography (CT)–guided biopsy of the mass revealed a high‐grade alveolar rhabdomyosarcoma (Figure 2). A positron emission tomography (PET) scan showed hypermetabolic activity of the large pelvic mass, along with areas in the lungs and ribs, concerning for metastatic disease (Figure 3). The clinical staging at this point was determined to be T2b, N1, and M1.

**Figure 4:**
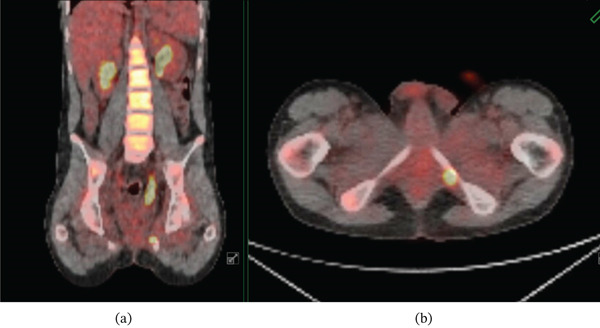
Select coronal (a) and axial (b) PET scan images demonstrating a residual 1.5‐cm area of hypermetabolic activity within the left obturator internus muscle despite multiple cycles of chemotherapy and radiation therapy.

Chemotherapy and radiation therapy (XRT) were initiated with the intention to treat without surgery as a wide resection would result in severe urinary dysfunction. The patient received neoadjuvant chemotherapy per our institutional protocol consisting of vincristine, dactinomycin, and cyclophosphamide, followed by maintenance therapy with vinorelbine and oral cyclophosphamide. The radiation therapy consisted of consolidated radiotherapy to the pelvis consisting of 33 fractions over 44 days with a total dose of 59.4 Gy. A PET scan 6 months after eight cycles of chemotherapy and 2 months of XRT showed excellent response except for a residual 1.5‐cm area with increased uptake in the obturator internus muscle, adjacent to the inferior pubic ramus. Despite continued chemotherapy, a repeat PET scan 9 months after the initiation of treatment showed continued uptake in the obturator internus which was concerning for viable tumor (Figure 4). Given the concern for persistent tumor activity, a wide resection was recommended and agreed upon by the patient, his family, and the multidisciplinary sarcoma team for definitive control.

## 3. Surgical Technique

**Figure 5:**
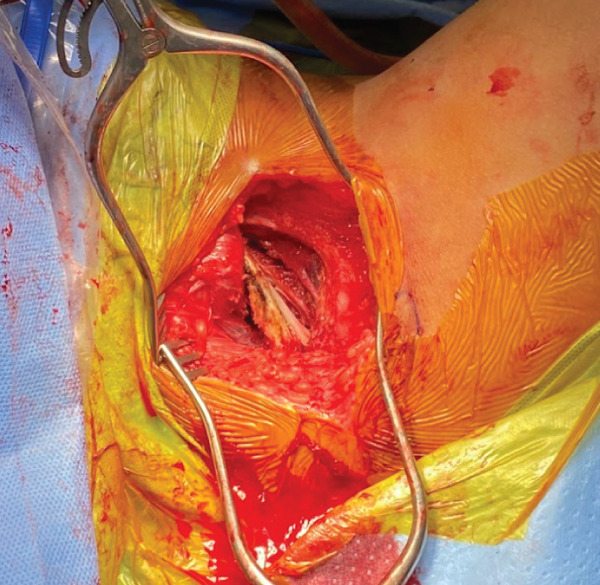
Intraoperative image of the inferior pubic ramus exposure with the perineal approach.

The patient was positioned in a lithotomy position. A 15‐cm longitudinal incision was made between the left ischial tuberosity and pubic body. The subcutaneous tissues and underlying fascia were carefully dissected in line with the skin incision. Care was taken to remain in one anatomic compartment and to avoid tissue planes. Meticulous hemostasis was maintained throughout the case. The inferior pubic ramus was encountered (Figure 5).

**Figure 6:**
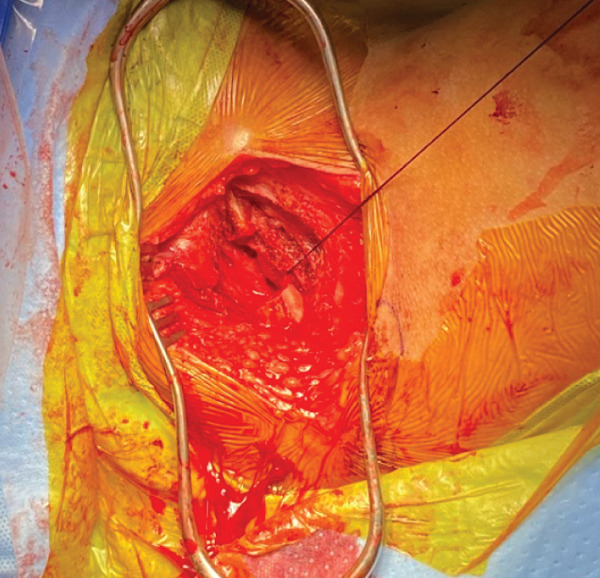
Intraoperative image of the inferior pubic ramus osteotomy to achieve better visualization of the pudendal nerve and mass (tagged with nylon suture) within the obturator internus muscle.

The mass was palpable just deep to the inferior pubic ramus; however, there was poor visualization around the bony structures. Due to limited visualization and the risk of damaging the pudendal nerve during dissection, an inferior pubic ramus osteotomy was performed at the medial and lateral obturator foramen (Figure 6). A 3‐cm segment of the inferior pubic ramus was removed. The pubic ramus was not felt to be involved in the tumor and was not treated as a surgical margin; it was removed for visualization and to increase access to deeper structures.

**Figure 7:**
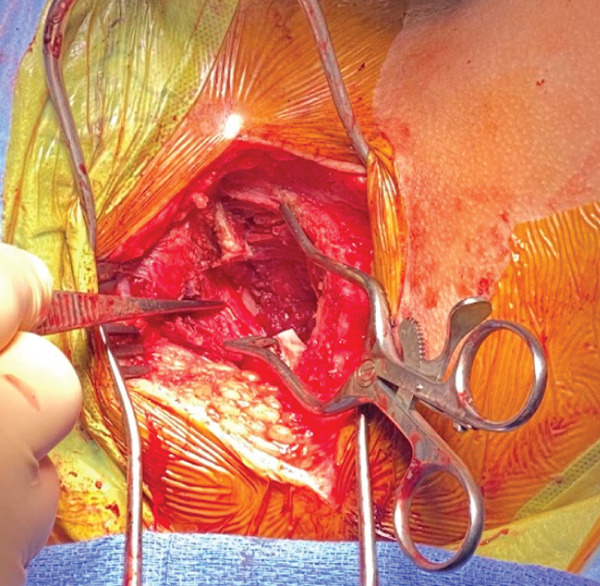
Intraoperative image of the pudendal neurovascular bundle.

Next, the interval between the obturator internus and obturator externus was carefully developed. Through the osteotomy, the mass and pudendal nerve were better visualized (Figure 7). The pudendal nerve was pushed anterior by mass effect from the tumor and was carefully dissected off of the anterior obturator internus and decompressed along the length of the muscle. The nerve was carefully protected for the remainder of the procedure. The mass in the obturator internus muscle was dissected with a cuff of normal tissue as a margin, circumferentially to its base, and removed in its entirety. No lymph nodes were submitted in the specimen.

**Figure 8:**
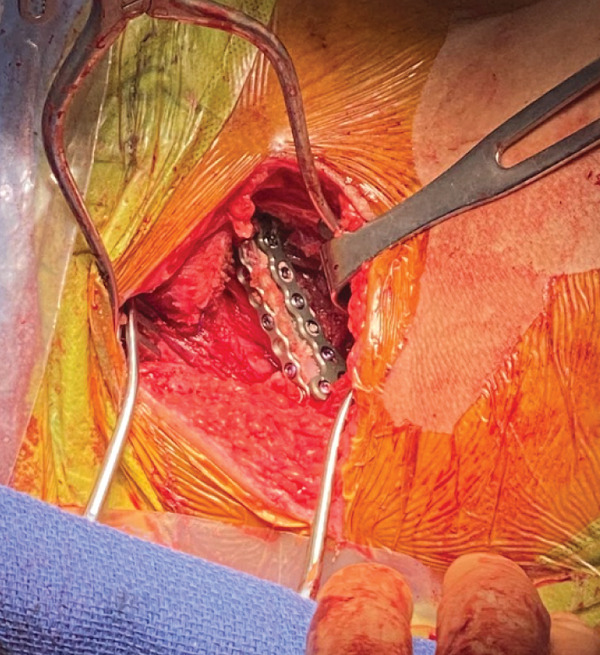
Intraoperative image of the inferior pubic ramus osteotomy fixation with orthogonal minifragment titanium plates.

The segment of the inferior pubic ramus was then replaced anatomically and fixed with orthogonal titanium minifragment plates, obtaining secure fixation of the bone fragment (Figure 8).

**Figure 9:**
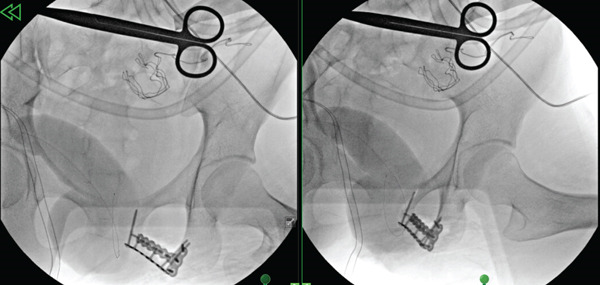
Intraoperative fluoroscopic images demonstrating fixation of the inferior pubic ramus osteotomy fragment.

**Figure 10:**
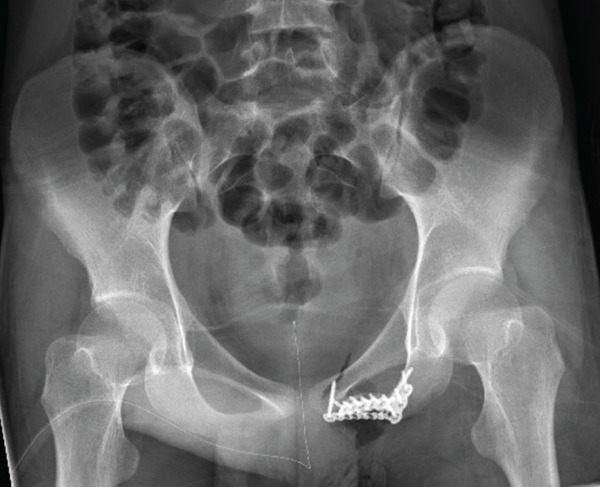
Anteroposterior (AP) pelvis radiograph demonstrating fixation of the inferior pubic ramus osteotomy fragment.

Appropriate implant position was confirmed on fluoroscopy (Figure 9). The wound was copiously irrigated and closed in layers. Sterile dressings were placed. A final radiograph was taken in the recovery area (Figure 10).

## 4. Follow‐Up

There were no postoperative complications. His incision and osteotomy healed without issue. He completed 6 weeks of maintenance chemotherapy and XRT to metastatic sites. The XRT consisted of whole lung XRT with 15 Gy over 10 fractions with a stereotactic beam radiation therapy (SBRT) boost to a rib lesion.

**Figure 11:**
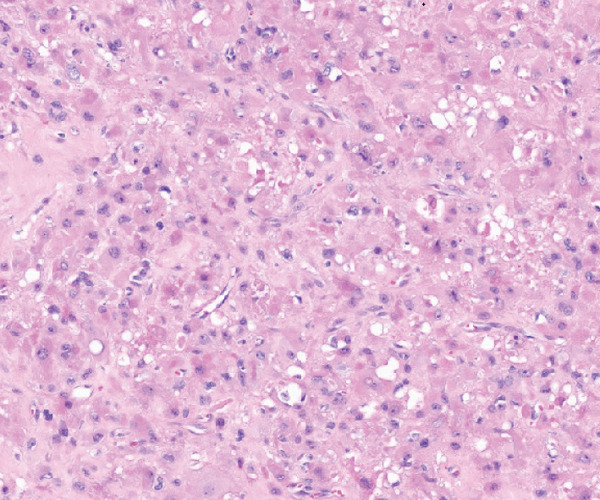
Medium power view (200X) showing rhabdomyoblasts with reduced nuclear atypia and decreased nucleus to cytoplasm ratio, consistent with treatment effect.

Microscopic assessment of the excised mass noted margins to be free of tumor (R0) with the closest margin being 8 mm. Pathology demonstrated cytodifferentiation with mature rhabdomyoblasts, consistent with treatment effect (Figure 11).

**Figure 12:**
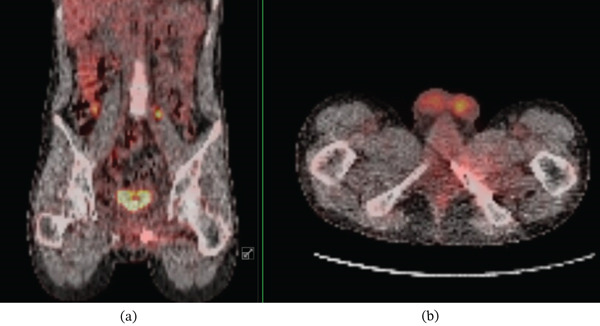
Select coronal (a) and axial (b) PET scan images demonstrating no activity in the previous hypermetabolic area in the obturator internus muscle.

A PET scan performed after the third cycle of maintenance chemotherapy showed no active disease (Figure 12). Postoperative rehabilitation consisted of a structured physical therapy program including progressive general strengthening, sport‐specific rehabilitation, and pelvic floor therapy aimed at restoring core stability, continence, and functional mobility.

Although formal patient‐reported outcome measures were not collected, clinical outcomes were assessed using functional and symptom‐based evaluations relative to the surgical site and anticipated morbidity.

At 1‐year follow‐up, he had returned to full participation in recreational sports without activity limitation. He reported normal urinary function without urgency, frequency, dysuria, or stress incontinence. Additionally, sexual function was preserved, and he denied any bowel dysfunction. Overall functional recovery was excellent, with the patient returning to his baseline level of activity and quality of life. He remains in remission 24 months following completion of neoadjuvant chemotherapy, surgical resection of the mass, and maintenance chemotherapy.

**Figure 13:**
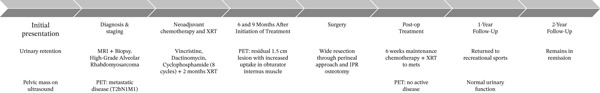
Summary of clinical timeline. MRI: magnetic resonance imaging; PET: positron emission tomography; XRT: radiation therapy; IPR: inferior pubic ramus.

Figure 13 summarizes the clinical timeline.

## 5. Discussion

Deep pelvic tumors adjacent to the inferior pubic ramus are rare and present significant surgical challenges due to complex pelvic anatomy and the high risk of damage to critical neurovascular and genitourinary structures. Our case describes a 17‐year‐old male with a large pelvic high‐grade alveolar rhabdomyosarcoma. He was treated with neoadjuvant chemotherapy and XRT with a persistent deep left pubic mass. Given the mass′s location in the obturator internus, we elected to use a perineal approach, first described by He et al. as the femoribus internus (inner thigh)–perineal approach for resection of inferior pubic ramus tumors [3].

The traditional ilioinguinal approach to this region is an anterior‐based approach that requires extensive dissection and has a greater risk of blood loss, damage to soft tissue or critical neurovascular structures, and postoperative complications [4–6]. Perineal approaches can be used for Enneking Type III pelvic resections of tumors that necessitate removal of the anterior pelvis [7, 8]. He et al. used a less invasive femoribus internus–perineal approach in a series of four patients to resect a variety of inferior pubic ramus tumors [3]. The approach provides direct access to the inferior pubic ramus and is associated with shorter operative times, reduced blood loss, and a lower risk of neurovascular injury. In the present case, this approach was used to resect a soft tissue mass adjacent to the inferior pubic ramus.

There is a paucity of literature on alternative surgical approaches for accessing the obturator internus muscle. Menge et al. described a minimally invasive medial approach, called the Vanderbilt approach, for drainage and debridement of the obturator musculature in pediatric patients [9]. Dissection is carried through the adductor brevis, following the anterior branch of the obturator nerve toward the central obturator foramen and medial ischium. Access to the obturator internus requires directing a clamp under fluoroscopic guidance through the obturator foramen and into the obturator internus. White et al. described a minimally invasive posterior transgluteal approach for debridement of an abscess within the obturator internus [10]. This approach involves splitting the gluteus maximus in line with the muscle fibers down to the ischial tuberosity. The obturator internus is accessed with blunt dissection on bone toward the medial ridge of the ischial tuberosity. Although both of these approaches allow for adequate drainage of an abscess, they would not provide enough exposure for the wide excision of a mass in the obturator internus muscle.

In our case, an inferior pubic ramus osteotomy was necessary to achieve adequate visualization and access to the mass. After the mass was resected, the bone fragment of the inferior pubic ramus was replaced anatomically and fixed with orthogonal titanium minifragment plates and screws. Although there are reports that describe no significant functional deficit with removal of this segment [11], our goal was to restore the native anatomy and preserve anterior pelvic ring stability in this young patient with high functional demands.

In the original description of the approach, the pudendal nerve was not encountered. The pudendal nerve is a critical structure to consider in this region, as it supplies innervation to the rectum, anus, perineum, and external genitalia. Additionally, it is responsible for the motor function of the external anal and urethral sphincters and plays a crucial role in sexual function [12]. Injury to the pudendal nerve may result in diminished sensation, sexual dysfunction, and fecal or urinary incontinence [13–15]. In our patient, the pudendal nerve was displaced anteriorly by the mass within the obturator internus muscle, placing it directly into the surgical field. Once the nerve was dissected along the length of the muscle, it was carefully retracted and protected for the remainder of the case.

Wide resection with a cuff of normal tissue was pursued to achieve oncologic control in accordance with established sarcoma principles, which prioritize complete excision with negative margins to minimize the risk of local recurrence. In this case, preoperative imaging localized the lesion to the obturator internus muscle, allowing for targeted excision of the involved muscle while preserving adjacent critical structures including the pudendal nerve and urogenital organs.

Finally, pathologic examination of the resected mass demonstrated cytodifferentiation with mature rhabdomyoblasts. This represents an uncommon scenario, as increased uptake on surveillance PET scans is most frequently associated with residual or recurrent disease in rhabdomyosarcoma. In our case, the persistent focal metabolic activity on serial imaging raised a high level of concern for residual viable tumor, particularly given the patient′s initial metastatic burden and the known prognostic implications of incomplete local control to merit surgical excision for definitive local control.

In the setting of rhabdomyosarcoma, achieving durable local control is critical, as residual disease is associated with increased risk of progression and decreased overall survival. Therefore, in cases where imaging findings remain suspicious, the threshold to pursue definitive management remains low. In consultation with a multidisciplinary sarcoma team, it was concluded that observation alone would carry a meaningful risk of undertreating persistent disease. Alternative diagnostic strategies were carefully considered. An incisional open biopsy would have carried a morbidity profile comparable to that of our planned resection given the deep pelvic location and need for a similar surgical exposure. A core needle biopsy, although less invasive, was deemed to carry a substantial risk of tumor bed contamination and sampling error, particularly in this anatomically complex region adjacent to critical neurovascular and urogenital structures. Accordingly, surgical resection was pursued as both a diagnostic and therapeutic intervention, should the lesion have represented recurrent sarcoma. This approach allowed for complete excision of the metabolically active lesion, elimination of any potential residual tumor burden, and obtaining definitive histopathologic assessment. Histopathologic confirmation excluded active disease and avoided the potential harm of undertreatment. This case highlights the limitations of PET imaging in isolation and underscores the critical importance of correlating imaging findings with clinical context, pathology, and multidisciplinary judgment when determining management in complex oncologic scenarios.

## 6. Conclusion

Our case expands on the limited literature on techniques for resecting tumors involving the obturator musculature. Using a perineal approach, we performed an inferior pubic ramus osteotomy and carefully preserved the pudendal nerve, successfully resecting a tumor in the obturator internus muscle with wide margins while minimizing functional deficits and urinary complications. This approach may represent a viable option in carefully selected patients when performed by an experienced multidisciplinary team.

## Funding

No funding was received for this manuscript.

## Ethics Statement

This study was conducted in accordance with Institutional Review Board (IRB) approval. This case report was prepared in accordance with the CARE guidelines.

## Consent

A written informed consent was obtained from the patient for this study and publication.

## Conflicts of Interest

The authors declare no conflicts of interest.

## Data Availability

Data sharing is not applicable to this article as no datasets were generated or analyzed during the current study.
